# Deep Learning Tool Wear State Identification Method Based on Cutting Force Signal

**DOI:** 10.3390/s25030662

**Published:** 2025-01-23

**Authors:** Shuhang Li, Meiqiu Li, Yingning Gao

**Affiliations:** School of Mechanical Engineering, Yangtze University, Jingzhou 434023, China; lsh1599634459@163.com (S.L.); gyn_gn@163.com (Y.G.)

**Keywords:** milling cutter status recognition, continuous wavelet transform, contextual transformer, global attention mechanism, MobileViT

## Abstract

The objective of this study is to accurately, expeditiously, and efficiently identify the wear state of milling cutters. To this end, a state identification method is proposed that combines continuous wavelet transform and an improved MobileViT lightweight network. The methodology involves the transformation of the cutting force signal during the milling cutter cutting process into a time–frequency image by continuous wavelet transform. This is followed by the introduction of a Contextual Transformer module after layer 1 and the embedding of a Global Attention Mechanism module after layer 2 of the MobileViT network structure. These modifications are intended to enhance visual representation capability, reduce information loss, and improve the interaction between global features. The result is an improvement in the overall performance of the model. The improved MobileViT network model was shown to enhance accuracy, precision, recall, and F1 score by 1.58%, 1.23%, 1.92%, and 1.57%, respectively, in comparison with the original MobileViT. The experimental results demonstrate that the proposed model in this study exhibits a substantial advantage in terms of memory occupation and prediction accuracy in comparison to models such as VGG16, ResNet18, and Pool Former. This study proposes an efficient identification method for milling cutter wear state identification, which can identify the tool wear state in near real-time. The proposed method has potential applications in the field of industrial production.

## 1. Introduction

The milling cutter, being the “teeth” of the milling machine tool, is susceptible to wear and failure during product processing. The wear condition of the milling cutter is a pivotal factor affecting product quality and machining efficiency [1]. Milling cutter failure can lead to machine tool failure and downtime, product quality variations, and even safety accidents. Research has demonstrated that tool wear-related downtime can reach up to 20%, a figure that significantly impacts productivity [2,3]. Panda et al. [4] have demonstrated that implementing a milling cutter condition monitoring system can reduce machining costs by a minimum of 30% during the production and machining of products. Consequently, the identification of milling cutter wear conditions is imperative in mitigating milling cutter failure, ensuring product quality, reducing downtime, enhancing productivity, and reducing production costs.

At present, the identification of milling cutter wear can be approached through two primary methods: direct identification and indirect identification. Direct identification involves empirical observation or the measurement of tool geometry to ascertain the present degree of wear [5]. Some scholars employ optical cameras or super depth-of-field microscopes to determine a more precise amount of tool wear. However, these methods incur high maintenance costs and are more cumbersome [6]. Conversely, indirect identification methods rely on the formulation of wear prediction formulas or models [3]. Noteworthy contributions include Bjerke et al.’s establishment of a cutting process wear model applicable to chemical wear, diffusion wear, and oxidation wear as predominant factors [7]. However, it should be noted that machining process interference can significantly impact model prediction accuracy [8], underscoring the limitations of physical models. At this stage, most of the research on milling tool wear state identification employs the indirect identification method of signal analysis. According to the established mapping between sensor signals and tool wear [9], state signals that are more pertinent to wear condition during milling are collected. These signals include, but are not limited to, cutting force signals [10,11], vibration signals [12,13], acoustic emission (AE) signals [14,15], spindle current signals, and tool tip temperature [16]. Subsequent to the collection of these signals, a process of feature extraction and analysis is initiated to ascertain the wear condition of the milling tool. Subsequently, the signals undergo a process of feature extraction, which enables the recognition of the tool’s health status.

Due to the presence of numerous interferences in the milling process, the monitored state signals frequently exhibit nonlinear and nonsmoothed characteristics. The current mainstream of feature extraction methods primarily encompasses time domain features, frequency domain features, and time–frequency domain features, categorized into three distinct classes. Time domain features and frequency domain features are primarily employed to characterize one-dimensional time series features, while time–frequency domain features are two-dimensional features of the signal, capable of depicting more subtle features and containing richer and more three-dimensional feature information. The most prevalent 2D time–frequency feature extraction methods include Fourier transform (FT), short-term Fourier transform (STFT), empirical modal decomposition (EMD), Hilbert transform, continuous wavelet transform (CWT), among others. Zhang et al. [17] collected and analyzed the acoustic emission signals during the operating state of the tool in the frequency and time–frequency domains by using Fourier transform and wavelet packet data decomposition (WPD). Guo et al. [18] analyzed the AE signals in the frequency and time–frequency domains by means of a generalized time–frequency distribution (TFD) obtained by horizontal synchronization transformation. This was used to extract the impulse characteristics of the tool’s unsteady vibration signals. The utilization of TFD results facilitates the detection of periodic impulses through two-dimensional Fourier transform. Huang et al. [19] proposed a tool wear monitoring method for vibration signals that employs short-term Fourier transform and a deep convolutional neural network (DCNN). The images of vibration signals were obtained by STFT, and the DCNN model was input to establish the relationship between time–frequency maps and tool wear states for wear state identification. While STFT is a viable method for 2D time–frequency map conversion, its fixed-width window is often inadequate in many scenarios. Narayana et al. [20] employed Empirical Mode Decomposition for the feature extraction of vibration signals and integrated it with Support Vector Machines (SVMs) to construct a multi-fault classification model. The classification accuracies of this method for the three types of faults. The accuracy rates were 94.4%, 94.2%, and 92.4%, respectively. Wang et al. [21] proposed an INGO optimization algorithm for the optimization of SVM hyperparameters, which achieved 97.9% identification accuracy for milling cutter wear state identification under the optimal parameter combination. Zhang et al. [22] employed the CWT, STFT, and Gramian angular summation field (GASF) to transform force signals into two-dimensional images for tool wear classification. The results indicated that the CWT-processed force signal state recognition model exhibited an accuracy rate exceeding 90%, surpassing the performance of the other two methods.

In the study of shallow machine learning state recognition models, T. Mikolajczyk et al. [23] employed an artificial neural network (ANN)-based image processing technique to estimate tool wear. The fundamental principle underlying this technique is to compute the width of the tool’s back face wear based on the number of pixels within the tool wear area. The experimental results demonstrated that, within the range of the tool’s lifetime, the average error between the results obtained by the method and the average error between the optical measurements is 6.7%. In an effort to develop a novel tool wear monitoring method, Wei et al. [24] based their approach on variational modal decomposition (VMD) and Hilbert–Huang Transformation (HHT). This method was utilized to monitor the wear of carbide tools during the machining of stainless steel. Li et al. [25] identified both normal and severe wear states of the milling tool at a rate higher than 95% based on a particle swarm-optimized SVM model. However, machine learning models are encumbered by a number of disadvantages, including a protracted training time, a multitude of setup parameters, and an inability to classify and recognize one-dimensional time series features. In recent years, an increasing number of scholars have employed deep learning models for recognizing the wear state of milling cutters. Marani M. et al. [26] demonstrated the efficacy of long short-term memory (LSTM) in tool wear recognition. Ma et al. [27] utilized Pearson’s correlation coefficient for extracting sensitive features from vibration signals and employed LSTM to predict tool wear amounts. Jiang et al. [28] designed a Generative Adversarial Network (GAN) for data enhancement purpose, which can reduce the amount of real environment industrial data required. Experimental results show that the proposed GAN helps to significantly improve the performance of tool wear classification models. The aforementioned models were enhanced for the temporal feature mining of data; however, there is considerable room for improvement with regard to the training duration and memory occupation of the model. MobileViT [29] is a lightweight generalized visual recognition model that combines the advantages of convolutional neural networks (CNNs) and Vision Transformer (ViT), with low latency, high accuracy, and better performance in achieving efficient image classification. In a comparative analysis conducted by Varam et al. [30], MobileViT was pitted against several lightweight deep network models. The evaluation metrics encompassed the F1 score, model size, and performance-to-size ratio. The findings of this study indicated that MobileViT exhibited the most optimal overall performance across the utilized dataset. Sun et al. [31] introduced the Focused Linear Attention (FL) mechanism in MobileViT, a development that led to a substantial reduction in computational complexity while maintaining the ASPP feature processing module. This integration enabled a more precise localization of tampered regions of various sizes. Xu et al. [32] combined the convolutional block attention module (CBAM) with the MV2 module and embedded the Bi-directional long short-term memory (BiLSTM) to construct the MobileViT-CBAM-BiLSTM model for the triple classification task of images, thereby enhancing the model’s generalizability.

According to the extant literature, the wear failure of milling cutters can result in product quality problems or even safety accidents. Consequently, the accurate and expeditious identification of milling cutter wear state is imperative. A synthesis of the extant research conducted by prominent scholars in the field reveals a robust correlation between the acquired milling cutter cutting force signal and the milling cutter wear state. However, the presence of noise interference necessitates the implementation of a feature extraction method to extract signal characteristics and mitigate the impact of interference. The basic depth model was shown to have a limited effect on the extraction of the two-dimensional feature map obtained after conversion. There is still some room for improvement in the overall recognition performance of the model. To address these limitations, this study proposes a novel method for recognizing the wear state of a milling cutter. The proposed approach utilizes the Continuous Wavelet Transform (CWT) and the MobileViT lightweight network to accurately and timely identify the wear stage of the tool. The primary objective of this method is to minimize defective products and safety accidents caused by tool wear. CWT, a potent time–frequency analysis instrument, facilitates the conversion of cutting force signals into two-dimensional time–frequency feature maps. This conversion process effectively mitigates the impact of noise and extraneous factors while preserving the local characteristics of the signals. The MobileViT network, distinguished by its lightweight and high efficiency, is employed to identify the wear state of the milling cutter. This integration of the Contextual Transformer (CoT) [33] and the Global Attention Mechanism (GAM) [34] enables the network to effectively recognize the wear state. The Global Attention Mechanism (GAM) modules were developed to enhance the model’s capacity to capture features of varying wear states while ensuring a low memory footprint and rapid recognition speed. This method was demonstrated to accurately and efficiently identify the current wear state of milling cutters, providing guidance for timely tool replacement. The method has a wide range of applications in industrial production. The remainder of this paper is structured as follows: Section 2 provides a comprehensive description of the overall recognition model proposed in this paper and the related techniques employed. The specific model parameter settings, evaluation metrics, and datasets used are described in Section 3. The subsequent section, 4, involves a comparative analysis of the underlying lightweight models. This comparative analysis forms the basis for the subsequent execution of ablation experiments on the modules and the subsequent analysis of the results. Finally, Section 5 offers a comprehensive summary of the proposed model’s strengths and limitations, along with directions for potential future enhancements. The proposed model aims to optimize the balance between accuracy, lightweight, and recognition speed, offering a novel approach for recognizing the tool wear state in real-time monitoring systems.

## 2. Signal Processing and Research Model

**Figure 1:**
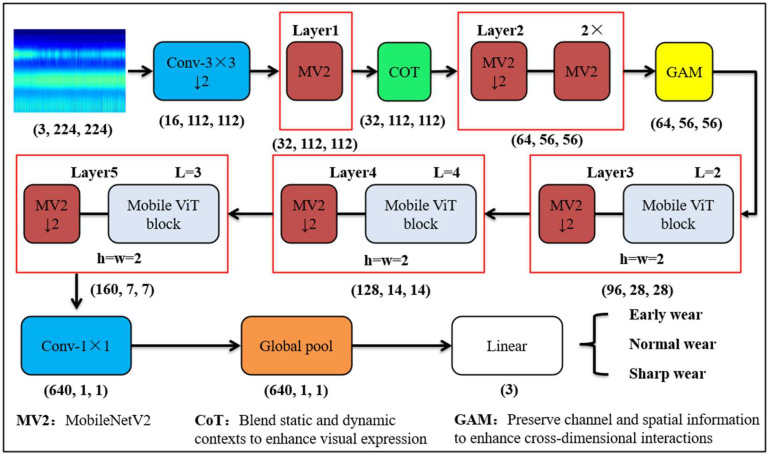
Diagram of milling cutter wear recognition model based on CWT and MobileViT.

Given the recognized impact of the milling process on the quality of the final product, it is imperative that the method of identifying the tool’s wear state be as accurate and efficient as possible. To address these demands, this paper proposes the utilization of the MobileViT lightweight deep learning network model as the base network. The cutting force signal collected during the milling process of the tool is transformed into a two-dimensional time–frequency diagram through continuous wavelet transform to serve as the input of the network. Concurrently, a CoT module and a GAM module are incorporated to enhance the extraction capability of the model for the features of different wear stages. Concurrently, the CoT module and the GAM module are incorporated into the network model to enhance its capacity to extract time–frequency diagram features, thereby optimizing the classification accuracy of the overall recognition model for distinct wear stages and improving the model’s overall performance. The overall model diagram is depicted in Figure 1.

The model utilizes the three-channel time–frequency map, sized 224 × 224, as the input for the network model. Through a 3 × 3 convolution with downsampling, the 16-channel feature map, sized 112 × 112, is obtained. Subsequently, the model traverses the modules of each layer of the network in turn. Ultimately, the 640 features are obtained through global pooling. These features are mapped into three classification output results after the fully connected layer. The classification results are encoded with One-Hot Encoding, consisting of [0, 0, 1] for early wear, [0, 1, 0] for normal wear, and [1, 0, 0] for sharp wear.

### 2.1. Continuous Wavelet Transform

Continuous wavelet transform is a type of time–frequency analysis method. CWT’s transformation principle involves determining a center frequency, acquiring a series of center frequencies and different intervals of basis functions through scale transformation and time shift change, and subsequently multiplying them one by one with the corresponding basis function intervals of the original signals. These signals are then integrated to obtain the frequency of the original signals within the specified interval. This method was shown to possess superior time–frequency localization characteristics and is adept at handling the analysis of non-stationary signal characteristics. It is less susceptible to noise interference, making it a suitable tool for a variety of applications, including equipment condition monitoring, fault diagnosis, life prediction, and medical monitoring. In this paper, CWT is employed to transform the one-dimensional original vibration signal into a two-dimensional time–frequency map, which contains time–frequency domain information. This transformation facilitates the analysis and processing of signal features by convolutional neural networks.

The CWT formula for an arbitrary signal $x\left(t\right)$ can be expressed as follows [35]:

(1) $$W_{\psi x\left(s,u\right)}=\left[x\left(t\right),\psi_{s,u}\left(t\right)\right]=\frac{1}{\sqrt{s}}\int_{-\infty }^{+\infty }x\left(t\right)\psi \left(\frac{t-u}{s}\right)dt$$

(2) $$\psi_{s,u}\left(t\right)=\frac{1}{\sqrt{s}}\psi \left(\frac{t-u}{s}\right)s,u\in R,s>0$$

In the aforementioned equation, the variable $\Psi_{s,u}\left(t\right)$ denotes the wavelet basis function, which is derived from the mother wavelet through a process of stretching and translation. The variable *s* is the scale factor, which modulates the dimensions of the wavelet window and its placement in the frequency domain. The variable *u* is the translation factor, which dictates the position of the wavelet window in the time domain. The wavelet basis function is employed to analyze the fundamental waveform of a signal, and the scale describes how much this fundamental waveform expands or contracts. Within the framework of the wavelet transform, an inverse relationship exists between scale and frequency. Consequently, smaller scales are indicative of higher frequencies, while larger scales correspond to lower frequencies. This property enables the wavelet transform to offer insights into the time domain and frequency domain characteristics of the signal. As the parameter *s* increases, the frequency of the wavelet function decreases, leading to a reduction in temporal resolution. Conversely, as *u* increases, the frequency of the wavelet function increases and the frequency resolution decreases.

**Figure 2:**
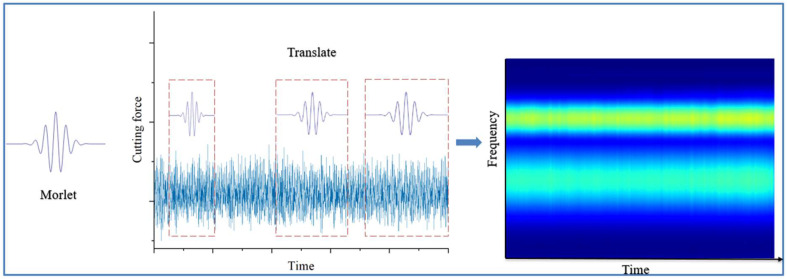
Schematic diagram of wavelet transform process.

In this paper, we analyze the milling cutter cutting force signal, which is characterized by nonsmoothness and nonlinearity. Najmi et al. [36] demonstrated that the Morlet wavelet function can provide good time and frequency resolution. Consequently, this paper proposes the utilization of the Morlet wavelet as the mother wavelet basis function for CWT processing of the cutting force signal. For the original cutting force signal, the non-cutting segment-specific signals were eliminated, and the last 100,000 rows of data collected from each milling were uniformly selected to be processed by CWT. This process was repeated to obtain the 224 × 224 two-dimensional time–frequency diagram. Given the nonsmooth and nonlinear characteristics of the cutting force signal, the Morlet wavelet basis function was selected. The signal sampling frequency was set to 50 kHz, resulting in each time–frequency diagram comprising 2 s of signal characteristic data. In the feature plot, the *X*-axis is time in seconds (s) and the *Y*-axis is frequency in hertz (Hz). Figure 2 provides a visual representation of the wavelet transform process, utilizing the milling cutter cutting force signal as a case study.

### 2.2. MobileViT

MobileViT integrates the lightweight design of Mobilenet with the attention mechanism of Vision Transformer, exhibiting high computational efficiency and a limited number of model parameters. The model is composed of a series of MobileNetV2 (MV2) modules [37] and MobileViT modules.

**Figure 3:**
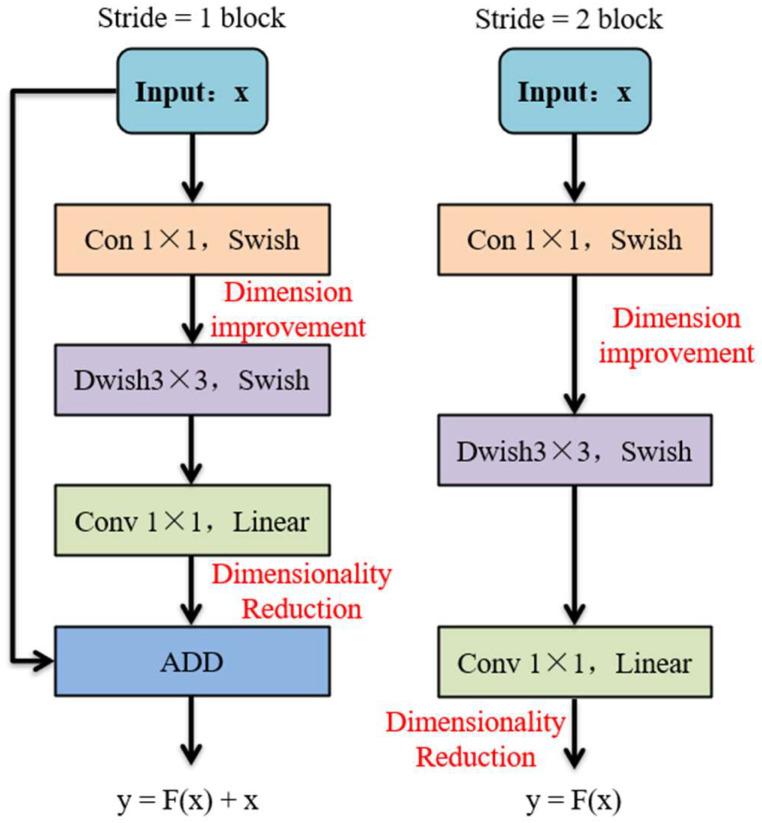
MV2 module structure.

The MV2 module employs a design structure that incorporates depth-separable convolution, inverted residual, and linear bottleneck, thereby effectively extracting local features while reducing the number of model parameters. The structural design of the MV2 module is illustrated in Figure 3.

Given that the height of the input feature matrix is *W* with *M* channels, the size of the convolution kernel is *K*, and the output feature matrix has *N* channels. The formulas for both conventional and separable convolution are summarized as follows.

(3) $$F_{1}=K\times K\times M\times N\times H\times W$$

(4) $$F_{2}=K\times K\times M\times H+M\times N\times H\times W$$

The MV2 module employs an inverted residual structure, which is distinct from the ResNet model’s approach of initially reducing the dimensionality and subsequently increasing it. The MV2 module utilizes a 1 × 1 point-by-point convolution to augment the dimensionality of the features, thereby enhancing their granularity. This is followed by a 3 × 3 convolution to ensure the completion of feature extraction for each channel. This approach leads to a reduction in the number of model parameters. Equation (3) indicates that the computational complexity of traditional convolution is considerably higher than that of depth-separable convolution. Consequently, the implementation of separable convolution can result in a substantial reduction in the number of computational parameters.

(5) $$\frac{F_{2}}{F_{1}}=\frac{1}{N}+\frac{1}{K^{2}}$$

Subsequent to the completion of the aforementioned steps, the features undergo a downsampling process through 1 × 1 point-by-point convolution, thereby reducing the model’s output dimension. To mitigate the potential loss of low-dimensional feature information triggered by the Relu activation function, the final 1 × 1 point-by-point convolution is substituted with a linear activation function. Residual linkage in the MV2 module is only applied to the specific model structure, satisfying the conditions that the step size is 1 and the input and output dimensions are equivalent. This prevents feature loss when the step size is 2, utilizing the serial linkage to downsample the feature layer.

**Figure 4:**
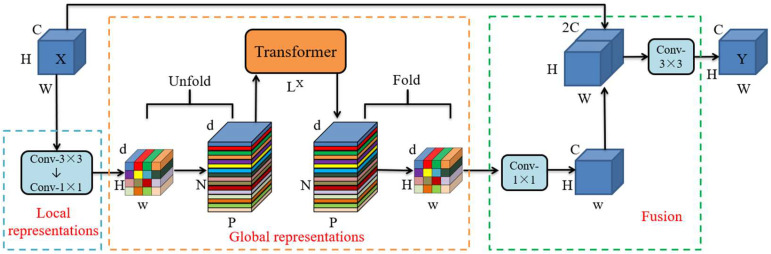
Structure of MobileViT module.

The MobileViT module represents a significant innovation in the field of machine learning, particularly in the context of transformer-based Vision Transformers. The module’s primary contribution lies in its integration of convolutions, a fundamental component of convolutional neural networks, with the transformer architecture found in ViTs. This integration results in the creation of a fusion module that exhibits both convolutional characteristics and the representation of global features, a feat that is crucial for achieving optimal performance in various vision tasks. The network structure diagram in Figure 4 illustrates its specific structure.

Given a feature map *X* of any size, the first step is to encode its local spatial information through a 3 × 3 convolutional layer. Then, a 1 × 1 convolutional layer is used to enhance the number of feature channels. Following this, the data are projected into the d-dimensional space (*d* > *C*), resulting in a reduction in the size of the feature map $X_{L}$ from *H* × *W* × *C* to *H* × *W* × *d*. To facilitate the model’s learning of global representation with spatial inductive bias, the feature map $X_{L}$ is subdivided into *N* non-overlapping image blocks of size h × w. Consequently, the image block sequence $X_{U}$ is of size *P* × *N* × *d*, where P=h×w, N=H×W/P. The relationships between image blocks in the sequence $X_{U}$ are then encoded by the Transformer for global information attention. To prevent the loss of information between image blocks, it is also necessary to restore the encoded image blocks to the pre-coding dimension to obtain the feature $X_{F}$. Finally, after the feature fusion module, the local features and global features are combined to obtain the output feature *Y* of this module.

### 2.3. Contextual Transformer

**Figure 5:**
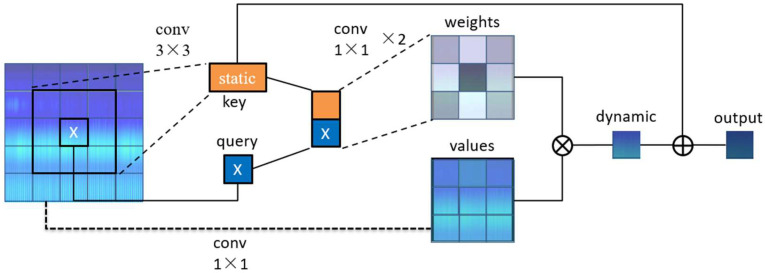
Structure of Contextual Transformer module.

The Contextual Transformer, a plug-and-play attention module proposed by Li et al. [33] in July 2021, has a structure that is illustrated in Figure 5.

The conventional approach primarily utilizes self-attention to derive the attention matrix, whereby keys and queries engage in a straightforward matrix multiplication. This method falls short in fully leveraging the contextual relationships between proximate keys. CoT delineates keys and queries as *K* = *X* and *Q* = *X*, respectively, and organizes and encodes the input keys through 3 × 3 convolutional blocks, thereby generating inputs in the static context representation form $K^{1}$. Subsequently, the encoded keys are combined with the input queries to learn the dynamic multi-head attention matrices ($W_{\delta }$ with ReLU activation function and $W_{\delta }$ without activation function) using two consecutive 1 × 1 convolutional blocks. The resulting matrices are multiplied by the input values, thus completing the dynamic context representation.

(6) $$A=\left[K^{1},Q\right]W_{\theta }W_{\delta }$$

Finally, the static and dynamic contexts are integrated into the final output of the module. This architecture integrates contextual information extraction between neighboring keys and the self-attentive learning of 2D feature maps into a single framework. Consequently, it eliminates the possibility of introducing additional branches that may be introduced due to mining between contexts. Overall, this improves the representational ability and overall performance of the network model.

### 2.4. Global Attention Mechanism

**Figure 6:**
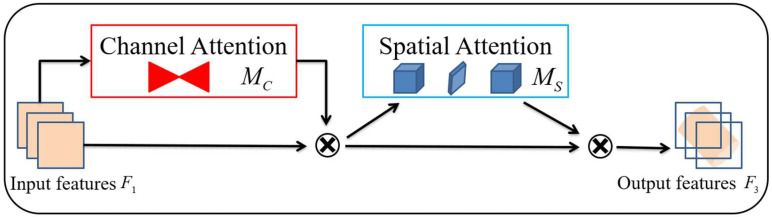
Structure of Global Attention Mechanism module.

The Global Attention Mechanism was proposed by Liu et al. [34] in October 2021. The original module of GAM is the sequential channel–space attention mechanism of CBAM. Sub-modules are redesigned and improved based on CBAM. The overall processing of feature map input by GAM is illustrated in Figure 6.

The location of the feature map is specified by $F_{1}$, which is denoted as RC × H × W. The intermediate state, designated as $F_{2}$, and the final output, denoted as $F_{3}$, are defined as follows:

(7) $$\begin{array}{l} F_{2}=M_{C}\left(F_{1}\right)⊗F_{1}\\ F_{3}=M_{S}\left(F_{2}\right)⊗F_{2} \end{array}$$

where $M_{C}$ is the channel attention map, $M_{S}$ is the spatial attention map, and ⊗ is the elemental multiplication.

The channel attention sub-module first preserves the 3D feature information by 3D alignment, followed by using a two-layer Multi-Perceptron Layer (MPL) to further amplify the channel–spatial dependencies across dimensions, resulting in stronger dependencies between the preserved 3D feature information.

The spatial attention sub-module’s primary function is to aggregate spatial information, and the main structure utilizes two 7 × 7 kernel convolutional layers to fuse the spatial information on a large scale. This process is based on the same channel attention sub-module reduction ratio as in the BAM module. The original module experiences a loss of feature information through the max-pooling layer, which can negatively impact the overall model performance. To mitigate this, the pooling layer is removed, preserving more comprehensive and detailed feature map information. However, this approach can potentially result in an increase in the model’s parameters in certain spatial attention module configurations.

## 3. Introduction to Experimental Setup and Dataset

### 3.1. Model Parameter Setting

The MobileViT training model utilized in the milling cutter wear dataset, which was constructed in this paper, has an iteration number of 1000. The number of training and validation batches per batch is 32, and the optimizer employs SGD, with a learning rate of 0.0125 and a weight decay coefficient of 0.0001. The loss function utilizes CrossEntropyLoss. The epoch is set to 1000, and the batch size is set to 32. This is a reference to the previous experience of setting the parameters of this model. The loss function is primarily employed to ascertain the discrepancy between the model’s actual output results and the sample labels. This discrepancy serves as feedback during back-propagation, facilitating the refinement of model parameters.

### 3.2. Model Evaluation Indicators

In the classification task, the loss value is defined as the distance between the predicted probability distribution and the true value. The cross-entropy loss function is typically employed to calculate the discrepancy, and its formula is expressed as follows:

(8) $$L=-\sum_{i=1}^{n}y_{i}\cdot \log{\hat{y}}_{i}$$

In this study, *n* is defined as the output category, $y_{i}$ is the true probability distribution, and ${\hat{y}}_{i}$ is the indicated predictive probability distribution.

The evaluation indexes related to model accuracy are used as accuracy, precision, recall, and F1 score with the following formula:

(9) $$Accuracy=\frac{TP+TN}{TP+TN+FP+FN}$$

(10) $$Precision=\frac{TP}{TP+FP}$$

(11) $$Recall=\frac{TP}{TP+FN}$$

(12) $$F1Score=2\cdot \frac{Precision\cdot Recall}{Precision+Recall}$$

Given that the milling cutter recognition model imposes specific constraints on the recognition time and the memory requirements of the model, subsequent comparisons of the base models will also utilize these metrics as indicators of model performance.

### 3.3. Introduction and Processing of Datasets

**Figure 7:**
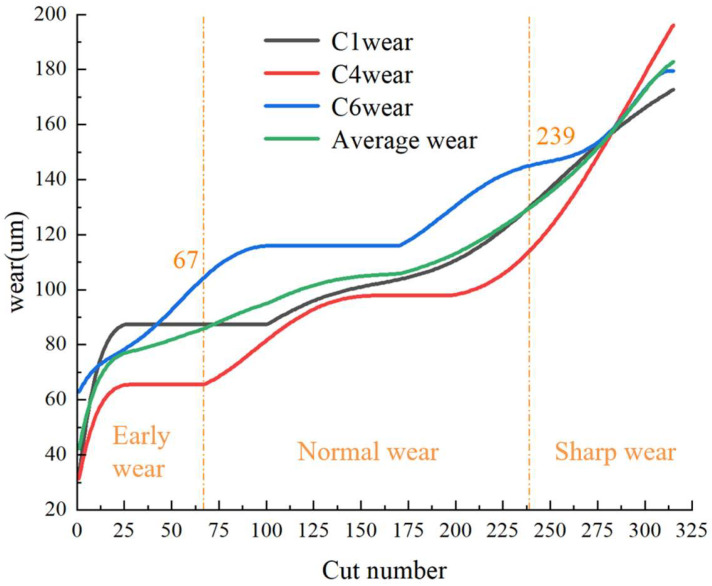
Wear stages of milling cutter.

The experimental dataset under consideration is the PHM2010 milling tool wear dataset. This dataset is a public dataset obtained from the Predictive and Health Management (PHM) Society, located in New York, USA, in the 2010 high-speed CNC machine tool competition [38]. The experiment involved the use of six milling cutters (C1~C6) to mill the workpiece sequentially, with a sampling frequency of 50 kHz. The wear on the back face of the cutters was measured at 10 min intervals using a microscope and recorded as a wear label for each sample. A total of 315 cuts were made on each tool. In this paper, three sets of experimental datasets with wear values recorded in C1, C4, and C6 are selected, resulting in 3 × 315 sets of data in total. This includes 630 sets for training and 315 sets for validation in each experiment. The reference standard employed in the present study aligns with the established practices documented in the extant literature [39]. The tool wear division under these conditions is illustrated in Figure 7.

**Figure 8:**
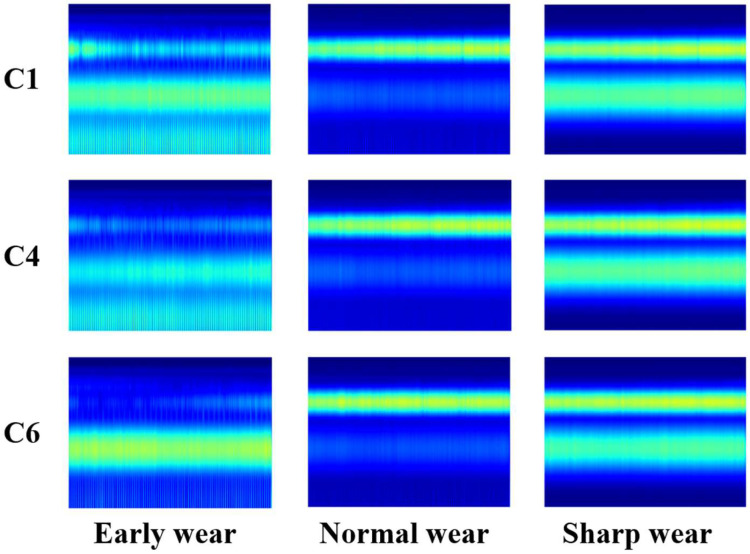
Continuous wavelet transform of each stage of milling cutter wear.

In Figure 7, the horizontal coordinate corresponds to the number of milling operations, while the vertical coordinate represents the tool wear value VB. The milling operations ranging from 0th to 67th are classified as the early wear stage, those from 68th to 238th as the normal wear stage, and those from 239th to 315th as the sharp wear stage. In this paper, we utilize the dataset comprising these three wear stages. Figure 8 below presents the continuous wavelet transform representative graphs.

## 4. Experimental Results and Discussion

### 4.1. Experimental Environment Configuration

The experimental hardware environment is composed of the following components: Intel(R) Xeon(R) Gold 5218, 128 GB RAM (Intel, Yingmai E-Commerce (Shanghai) Co., Ltd., Shanghai, China), and GPU: NVIDIA GeForce RTX 3090 (NVIDIA, Shenzhen Colorful Yugong Technology and Development Co., Ltd., Shenzhen, China) graphics card. The software operating environment is based on the Windows operating system, and the programming language is Python 3.6.13, CUDA version 11.0. The deep learning framework uses Pytorch 1.7.1.

### 4.2. Comparison of Basic Network Models

**Figure 9:**
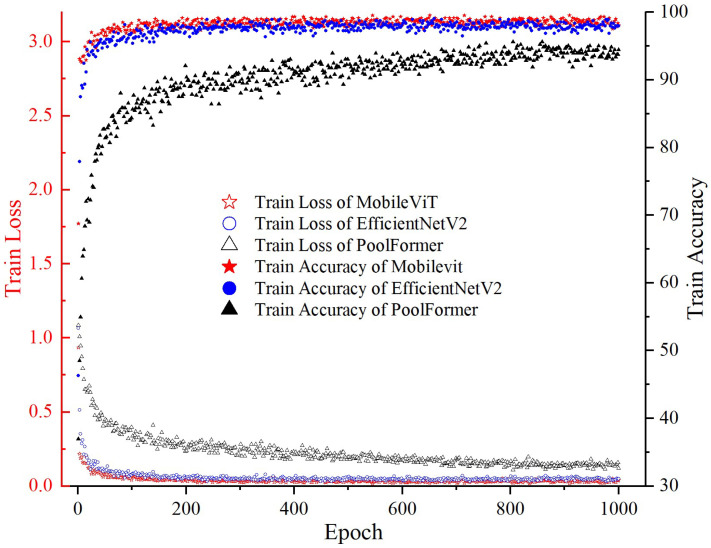
Training loss and accuracy.

**Figure 10:**
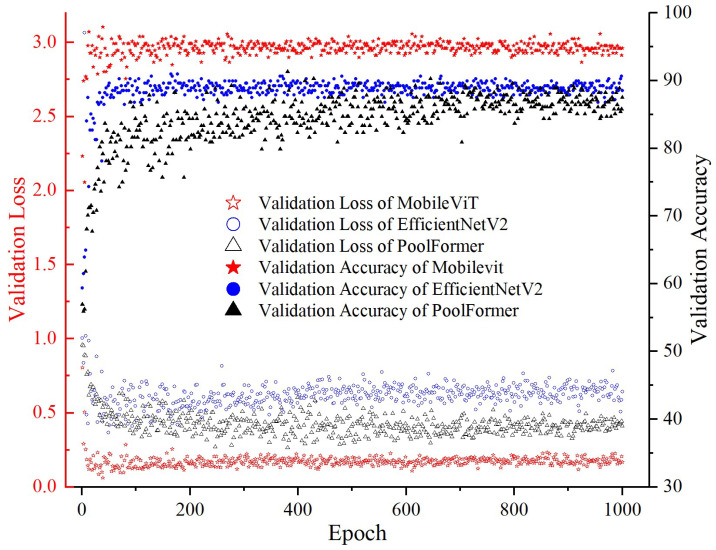
Verification loss and accuracy rate.

In order to meet the requirements of timeliness, accuracy, and lightweight of the milling cutter wear state recognition, a comparison is made of some of the more frequently used basic models. Among them, Vision Transformer and Pool Former exhibit a similar process, and the trend of the remaining models is roughly the same. To circumvent graphical redundancy, the training processes of Pool Former, EfficientNetV2, and MobileViT are selected for comparison and demonstration (Figure 9 and Figure 10). The comprehensive model comparison results are tabulated in Table 1.

As illustrated in Figure 9 and Figure 10, the training process of the model represented by Pool Former demonstrates a gradual convergence, reaching a steady state after approximately 800 epochs. The model represented by EfficientNetV2 exhibits higher training loss and validation loss compared to MobileViT in the initial stage, and the validation loss of EfficientNetV2 remains the highest after stabilization. In contrast, MobileViT stabilizes within approximately 200 epochs, exhibiting minimal fluctuations in both the training and validation processes.

As illustrated in Table 1, VGG16 demonstrates the highest recognition accuracy on the test set, MobileViT exhibits the smallest memory footprint, and EfficientNetV2 requires the least time to recognize a single image. However, VGG16’s recognition model memory occupation is as high as 1024 MB, compared to MobileViT, and its accuracy is only improved by 0.45%, but the memory occupation is increased by 26 times; when EfficientNetV2 is compared to MobileViT, its recognition time is only 3.17 ms faster, which is not very influential in the actual work, but its accuracy and memory occupation do not have the advantage. Therefore, given the three indicators of accuracy rate, memory usage, and recognition time, as well as the trend of training and validation, this paper elects to employ MobileViT as the foundational network model for the ensuing research.

### 4.3. Selection of Attention Modules and Analysis of Model Results

The Mobilevit-CoT-GAM model is predicated on the MobileViT base network model, with the Contextual Transformer module and the Global Attention Mechanism module embedded after layers 1 and 2, respectively. The following ablation experiments are designed to verify the effect of these modules in different positions of the model on the overall performance of the model. The results of the ablation experiments are shown in Table 2. In cases where a module is not added, the value “-” is indicated. The modularized Split-Attention block is represented by “ResNeSt [40]”.

As indicated by the findings presented in Table 2, the incorporation of ResNeSt, GAM, or CoT following layer 1 can enhance the model’s recognition accuracy to a certain extent. Conversely, the exclusive embedding of the GAM module after layer 2 results in a marginal enhancement of the model’s recognition accuracy. However, the embedding of ResNeSt and CoT modules leads to a decline in the recognition accuracy of the original model. This phenomenon can be attributed to the fact that the feature map in layer 1 is processed exclusively by a standard MV2 module, which does not alter the dimensions of the feature map. Consequently, the feature map retains a significant proportion of the characteristics of the initial input, enabling the embedded modules to capture these features more effectively. Conversely, layer 2 implements a 2× downsampling MV2 module, which modifies the spatial dimension and the number of feature channels of the feature map, while also reducing the width and height of the feature map to half of the original. This results in the skipping of some pixels, leading to the loss of information regarding the small differences contained within the feature map. Consequently, the embedding module is unable to capture the features present in these pixels. Finally, the CoT and ResNeSt modules, which are deficient in overall spatial and channel feature processing and prioritize contextual feature linking and cleavage stacking of feature maps, demonstrate substandard performance in comparison to the original model. In contrast, the GAM module, which emphasizes the integration of channel and spatial feature extraction, is capable of capturing subtle variations in the feature maps at each stage to a certain extent. Therefore, there is a marginal enhancement in the performance of the model.

A comparison of the results obtained from the integration of the embedded modules following layers 1 and 2 reveals that the combination of the GAM after layer 1 and the ResNeSt module in layer 2 exhibits the poorest performance in comparison to the original model. Conversely, the integration of the CoT after layer 1 and the GAM module in layer 2 demonstrates the most optimal performance in relation to the original model. The aforementioned results are attributable to the following sequence of events. Initially, the GAM module is embedded in layer 1 to capture the channel and spatial features. Subsequently, the feature map obtained undergoes 2-fold downsampling via the MV2 module in layer 2. This results in the features in the multi-dimensional feature map, which contain dense information integrated by the GAM module, becoming more compact. Subsequently, the feature map is split by the ResNeSt module. Subsequently, the feature map group undergoes subdivisions and weighting through the implementation of attention blocks, which results in a loss of the original features and, consequently, impacts the model’s overall recognition performance. The CoT module, situated after layer 1, has the capacity to emphasize the rich context information present within feature maps while maintaining their original dimensions. It integrates the static context with the dynamic context, facilitating more precise capture of subtle variations in feature maps. Subsequently, the 2-fold downsampling MV2 module in layer 2 employs a reduction in width and height by half, thereby enhancing the enrichment of detailed features within feature maps. Concurrently, the GAM module retains both the multi-channel features and the spatial information features to enhance the cross-dimensional interaction. The GAM module then aggregates and organizes the features to ultimately improve the overall recognition performance of the model. A comparison of the results of the module ablation experiments indicates that the model’s accuracy, precision, recall, and F1 scores are enhanced by 0.63 to 2.54, 0.41 to 2.63, 0.93–2.59, 0.70–2.58, respectively, after the placement of CoT in layer 1 and the embedding of GAM in layer 2. The improvements in F1 scores were found to be significant, with increases of 1.58%, 1.23%, 1.92%, and 1.57%, respectively, compared to the original MobileViT model. The findings indicate that the combination of CoT and GAM yields the most pronounced synergistic enhancement effect on the original model, thereby substantiating the efficacy of the improvement measures proposed in this study.

### 4.4. Improvement of Modeling Performance for Identifying Milling Cutter Wear States

**Figure 11:**
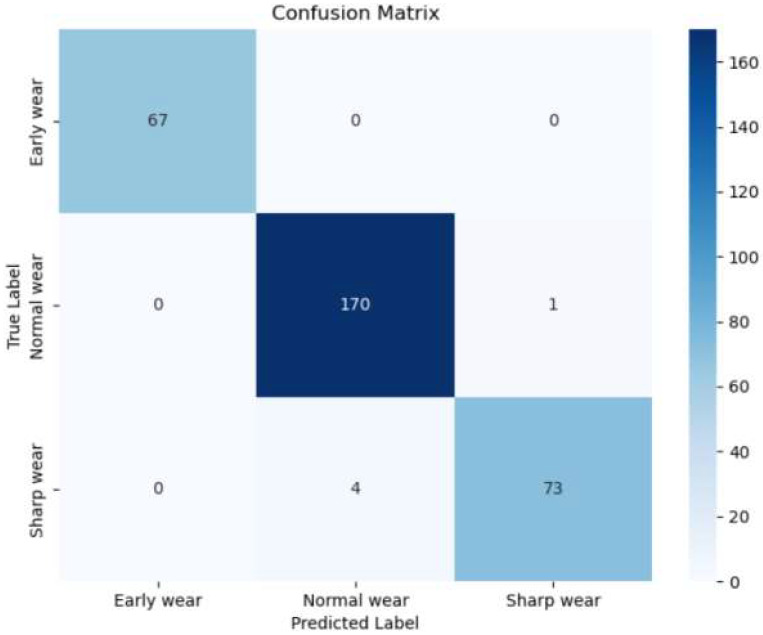
Confusion matrix for model identification of milling cutter wear stages.

In order to facilitate a more intuitive understanding of the performance of the proposed model in the task of milling cutter wear state recognition, the table of classification accuracies of the model for the three types of labels (Table 3) and the confusion matrix (Figure 11) are selected as a demonstration in this paper.

**Figure 12:**
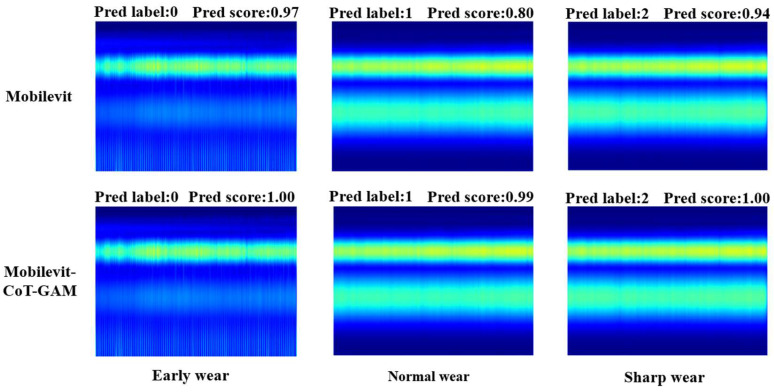
Comparison of single image prediction at each stage.

The aforementioned results indicate that the model’s accuracy in recognizing image features from the test set of 67 wear stages is 100%. This is attributed to the early wear stage of the feature map in the time–frequency domain, which manifests as more pronounced high-frequency signal characteristics. The feature map of the signal band exhibits relative concentration, facilitating effective extraction and identification of features. In the test set, 4 out of 174 images with normal wear stage are recognized as acute wear stage, and their recognition accuracy is 97.70%; 1 out of 74 images with acute wear stage is recognized as normal wear stage, and the recognition accuracy is 98.65%. In both the normal wear stage and the sharp wear stage, the presence of tool wear leads to the manifestation of features in both the high-frequency band color band and the low-frequency band. The feature map of several milling signals at the point of phase division exhibits a high degree of similarity, resulting in a certain degree of confusion in the recognition model when extracting similar feature information. To address this challenge, this paper proposes a novel approach for validating the input feature maps in the vicinity of the wear region. Specifically, the MobileViT model and the CoT-GAM-MobileViT are selected for comparison, and the results of this comparison are presented in Figure 12.

The 65th milling feature map in early wear, the 232nd milling feature map in normal wear, and the 250th milling feature map in sharp wear were selected from the above images for single image classification and recognition using MobileViT and CoT-GAM-MobileViT, respectively. The results demonstrate that both models can accurately identify the current wear stage of the tool. The precision scores of the MobileViT recognition model in the three stages are 0.97, 0.80, and 0.94, respectively, and the precision scores of the CoT-GAM-MobileViT recognition model in the three stages are 1.00, 0.99, and 1.00, respectively. Improved models: In the three stages of wear recognition, the recognition accuracy is higher than that of the original model, which more intuitively demonstrates the effectiveness of the improved model.

In order to further underscore the merits of the proposed method, a side-by-side comparison was conducted between the present study and two existing works. The results of this comparison are presented in Table 4.

Two research articles were selected for analysis; these articles also use the PHM2010 dataset to classify tool wear states. The accuracy, precision, and recall values of these articles were used as evaluation metrics to compare with the model proposed in this paper. The findings indicate that our model exhibits a recall of 98.07%, which is the highest among the three models, 3.77% higher compared to the literature [21] and 10.29% higher compared to the literature [22]. This finding signifies that the proposed model in this study demonstrates the most effective performance in identifying positive samples. This is particularly significant for tool wear monitoring, as a high recall reduces the occurrence of missed detections. In addition, the model under scrutiny exhibited superior performance in terms of accuracy and precision when compared to the other two models. This finding suggests that the model in question demonstrates a more robust overall performance in the domains of feature extraction and the classification of feature maps.

## 5. Conclusions

Considering the noise interference inherent in the collected cutting force signal and the potential impact of milling cutter wear on product quality and safety, we have incorporated the following criteria into our methodology: recognition accuracy, recognition time, and a lightweight prediction model. This paper proposes a milling cutter wear state recognition method by embedding CoT and GAM modules into MobileViT. This method effectively realizes accurate, fast, and low memory occupation recognition of the three wear phases on the open dataset. The primary conclusions of this study are outlined as follows:

(1) A model for identifying the wear state of milling cutters is proposed. This model is based on the transformation of the signal into a two-dimensional spectrogram as model input using continuous wavelet transform. To the best of the authors’ knowledge, this is the first time that CoT and GAM modules were embedded in the MobileViT lightweight network for milling cutter wear state identification.
(2) The cutting force signal feature map that was processed by continuous wavelet transform (CWT) is utilized as the model input. The overall performance of the model is comprehensively evaluated by three indexes: accuracy rate, model memory occupation, and single image recognition time. The comparison indicates that MobileViT exhibits optimal overall performance.
(3) The findings of the ablation experiments demonstrate that the CWT-MobileViT-CoT-GAM model enhances accuracy by 1.58%, precision by 1.23%, recall by 1.92%, and F1 score by 1.57%, respectively, in comparison to the original MobileViT model. Furthermore, the enhanced model exhibits a more substantial enhancement in overall performance.

In summary, the CWT-Mobilevit-CoT-GAM model proposed in this paper enhances the accuracy of the three stage identification of milling cutter wear, improves the generalization ability, and enhances the overall performance of the model. The method’s primary function is to accurately and efficiently identify the wear state of the milling cutter. This identification can serve as a reference for the timely replacement of the tool, thereby reducing the number of defective products resulting from the milling cutter’s rapid wear. Additionally, it can mitigate safety hazards associated with prolonged rapid wear and chipping. The model’s application in ensuring the quality and safety of operators is a notable benefit. However, the model does exhibit certain deficiencies, which will be explored and improved in subsequent sections. Firstly, the utilization of a public dataset for training and validation introduces a certain degree of generalization deficiency. Secondly, the restriction to cutting force signals in this study results in a relative paucity of information features in the feature map. Finally, the paper’s proposal of a wear state identification method is not accompanied by a corresponding upper computer monitoring software interface, which would facilitate user observation and use. In light of the aforementioned three deficiencies, the following research is hereby proposed: (1) The collection of additional datasets of varying specifications and materials for model training through experimentation, with further adoption of migration learning to enhance the model’s generalization performance. (2) The transformation of multi-dimensional signals into feature maps, followed by weighted fusion as the input of the wear recognition model, is imperative. This approach ensures the effective and comprehensive application of indirectly collected signals to the training of the recognition model. (3) Conduct research on the prediction and life of tool wear and develop a corresponding software interface for monitoring the upper computer to enhance the practicality of the method.

## Figures and Tables

**Table 1:** Comparison of performance of different base models.

Model	Accuracy	Memory Usage	Identification Time
VGG16	97.28%	1024 MB	28.57 ms
ResNet18	95.14%	85.9 MB	34.92 ms
Vision Transformer	95.73%	1003.52 MB	26.98 ms
EfficientNetV2	90.53%	155 MB	23.28 ms
Pool Former	88.69%	348 MB	27.35 ms
MobileViT	96.83%	38.2 MB	26.45 ms

**Table 2:** Results of ablation experiments.

Number	Layer 1	Layer 2	Accuracy	Precision	Recall	F1 Score
1	-	-	96.83%	97.55%	96.15%	96.84%
2	ResNeSt	-	97.46%	98.22%	96.71%	97.42%
3	-	ResNeSt	96.51%	97.66%	95.48%	96.39%
4	GAM	-	97.46%	98.17%	96.58%	97.33%
5	-	GAM	97.14%	98.03%	96.34%	97.10%
6	CoT	-	97.78%	98.37%	97.14%	97.71%
7	-	CoT	96.51%	97.34%	95.58%	96.40%
8	ResNeSt	GAM	97.14%	98.33%	96.04%	97.07%
9	ResNeSt	CoT	96.83%	97.26%	96.32%	96.67%
10	GAM	ResNeSt	95.87%	96.15%	95.56%	95.83%
11	GAM	CoT	97.14%	98.01%	96.34%	97.07%
12	CoT	ResNeSt	96.51%	97.06%	95.95%	96.44%
13	CoT	GAM	98.41%	98.78%	98.07%	98.41%

**Table 3:** Classification accuracy for each stage of milling cutter wear.

Classes	Precision	Recall	F1 Score	Average Precision
Early wear	100.00%	100.00%	100.00%	100.00%
Normal wear	97.70%	99.42%	98.55%	99.76%
Sharp wear	98.65%	94.81%	96.69%	99.17%

**Table 4:** A comparison of the literature.

	Accuracy	Precision	Recall
Ref. [21]	97.90%	98.60%	94.30%
Ref. [22]	>90%	92.93%	87.78%
This paper	98.41%	98.65%	98.07%

## Data Availability

Data are contained within the article.

## References

[1] del Olmo A., López de Lacalle L.N., de Pissón G.M., Pérez-Salinas C., Ealo J.A., Sastoque L., Fernandes M.H. (2022). Tool wear monitoring of high-speed broaching process with carbide tools to reduce production errors. Mech. Syst. Sign. Process..

[2] Yang Y., Guo Y., Huang Z., Chen N., Li L., Jiang Y., He N. (2019). Research on the milling tool wear and life prediction by establishing an integrated predictive model. Measurement.

[3] Zhou Y., Xue W. (2018). Review of tool condition monitoring methods in milling processes. Int. J. Adv. Manuf. Technol..

[4] Panda A., Nahornyi V., Valíček J., Harničárová M., Kušnerová M., Baron P., Pandová I., Soročin P. (2022). A novel method for online monitoring of surface quality and predicting tool wear conditions in machining of materials. Int. J. Adv. Manuf. Technol..

[5] Wang J., Xie J., Zhao R., Zhang L., Duan L. (2017). Multisensory fusion based virtual tool wear sensing for ubiquitous manufacturing. Robot. Comput.-Integr. Manuf..

[6] Dutta S., Pal S.K., Mukhopadhyay S., Sen R. (2013). Application of digital image processing in tool condition monitoring: A review. CIRP J. Manuf. Sci. Technol..

[7] Bjerke A., Hrechuk A., Lenrick F., Markström A., Larsson H., Norgren S., M’Saoubi R., Björk T., Bushlya V. (2021). Thermodynamic modeling framework for prediction of tool wear and tool protection phenomena in machining. Wear.

[8] Martínez-Arellano G., German T., Svetan R. (2019). Tool wear classification using time series imaging and deep learning. Int. J. Adv. Manuf. Technol..

[9] Mohanraj T., Shankar S., Rajasekar R., Sakthivel N.R., Pramanik A. (2020). Tool condition monitoring techniques in milling process—A review. J. Mater. Sci. Technol..

[10] Sahoo P., Patra K., Singh V.K., Mittal R.K., Singh R.K. (2020). Modelling Dynamic Stability and Cutting Forces in Micro Milling of Ti6Al4V using Intermittent Oblique Cutting FEM Simulation-based Force Coefficients. J. Manuf. Sci. Eng.—Trans. ASME.

[11] Yang X., Yuan R., Lv Y., Li L., Song H. (2022). A novel multivariate cutting force-based tool wear monitoring method using one-dimensional convolutional neural network. Sensors.

[12] Kiew C.L., Brahmananda A., Islam K.T., Lee H.N., Venier S.A., Saraar A., Namazi H. (2020). Complexity-based analysis of the relation between tool wear and machine vibration in turning operation. Fractals.

[13] Duan J., Shi T., Zhou H., Xuan J., Wang S. (2021). A novel ResNet-based model structure and its applications in machine health monitoring. J. Vib. Control.

[14] Huang W., Li Y., Wu X., Shen J. (2023). The wear detection of mill-grinding tool based on acoustic emission sensor. Int. J. Adv. Manuf. Technol..

[15] Liu J., Jiang C., Jiang H., Jiang Z. (2024). Optimizing tool life in SiCp/Al composites milling with acoustic emission analysis: A comprehensive monitoring and implementation strategy. J. Manuf. Process..

[16] Rizal M., Ghani J.A., Nuawi M.Z., Haron C.H.C. (2017). Cutting tool wear classification and detection using multi-sensor signals and Mahala Nobis-Taguchi System. Wear.

[17] Zhang C., Wang J., Cao Y., Jiao F. (2024). Tool wear status monitoring under laser-ultrasonic compound cutting based on acoustic emission and deep learning. J. Mech. Sci. Technol..

[18] Guo M., Tu X., Abbas S., Zhuo S., Li X. (2024). Time-frequency analysis-based impulse feature extraction method for quantitative evaluation of milling tool wear. Struct. Health Monit..

[19] Huang Z., Zhu J., Lei J., Li X., Tian F. (2021). Tool wear monitoring with vibration signals based on short-time Fourier transform and deep convolutional neural network in milling. Math. Probl. Eng..

[20] Narayana Moorthy N., Kanish T.C. (2022). Fault detection and identification in friction drilling process. J. Tribol..

[21] Wang J., Xiang Z., Cheng X., Zhou J., Li W. (2023). Tool wear state identification based on SVM optimized by the improved northern goshawk optimization. Sensors.

[22] Zhang Y., Qi X., Wang T., He Y. (2023). Tool wear condition monitoring method based on deep learning with force signals. Sensors.

[23] Ajczyk T.M., Nowicki K., Odowski A.K., Pimenov D.Y. (2017). Neural network approach for automatic image analysis of cutting-edge wear. Mech. Syst. Signal Process..

[24] Wei W., He G., Yang J., Li G., Ding S. (2023). Tool Wear Monitoring Based on the Gray Wolf Optimized Variational Mode Decomposition Algorithm and Hilbert–Huang Transformation in Machining Stainless Steel. Machines.

[25] Li J., Lu J., Chen C., Ma J., Liao X. (2021). Tool wear state prediction based on feature-based transfer learning. Int. J. Adv. Manuf. Technol..

[26] Marani M., Zeinali M., Songmene V., Mechefske C.K. (2021). Tool wear prediction in high-speed turning of a steel alloy using long short-term memory modelling. Measurement.

[27] Ma K., Wang G., Yang K., Hu M., Li J. (2022). Tool wear monitoring for cavity milling based on vibration singularity analysis and stacked LSTM. Int. J. Adv. Manuf. Technol..

[28] Jiang Y., Drescher B., Yuan G. (2023). A GAN-based multi-sensor data augmentation technique for CNC machine tool wear prediction. IEEE Access.

[29] Mehta S., Rastegari M. (2021). Mobilevit: Light-weight, general-purpose, and mobile-friendly vision transformer. arXiv.

[30] Varam D., Khalil L., Shanableh T. (2024). On-Edge Deployment of Vision Transformers for Medical Diagnostics Using the Kvasir-Capsule Dataset. Appl. Sci..

[31] Sun J., Zhang F., Liu H., Hou W. (2024). Research on Improved MobileViT Image Tamper Localization Model. Comput. Mater. Contin..

[32] Xu L., Huang Z., Long W., Jiang L., Tong X. (2024). Classification Model of Fish Feeding Intensity Based on MobileViT-CBAM-BiLSTM. Trans. Chin. Soc. Agric. Mach..

[33] Li Y., Yao T., Pan Y., Mei T. (2022). Contextual transformer networks for visual recognition. IEEE Trans. Pattern Anal. Mach. Intell..

[34] Liu Y., Shao Z., Hoffmann N. (2021). Global attention mechanism: Retain information to enhance channel-spatial interactions. arXiv.

[35] Berger B.S., Minis I., Harley J., Rokni M., Papadopoulos M. (1998). Wavelet based cutting state identification. J. Sound Vib..

[36] Najmi A.-H., Sadowsky J. (1997). The continuous wavelet transform and variable resolution time-frequency analysis. Johns Hopkins APL Tech. Dig..

[37] Sandler M., Howard A., Zhu M., Zhmoginov A., Chen L.C. Mobilenetv2: Inverted residuals and linear bottlenecks. Proceedings of the IEEE Conference on Computer Vision and Pattern Recognition.

[38] The Prognostics and Health Management Society 2010 PHM Society Conference Data Challenge. https://www.phmsociety.org/competition/phm/10.

[39] Zhu K., Zhang Y. (2019). A generic tool wear model and its application to force modeling and wear monitoring in high speed milling. Mech. Syst. Signal Process..

[40] Zhang H., Wu C., Zhang Z., Zhu Y., Lin H., Zhang Z., Sun Y., He T., Mueller J., Manmatha R. Res: Split-attention networks. Proceedings of the IEEE/CVF Conference on Computer Vision and Pattern Recognition.

